# 

**Published:** 1880-12

**Authors:** 


					To Readers and Correspondents.
Communications intended for publication, and exchange journals and pa-
pers, should be addressed to the Editor of the American Journal of. Den-
tal Science, 259 N. Eutaw St., Baltimore, Md. All letters relating to the
business of the journal, or containing cash remittances, to be directed to
Messrs. Snowden & Cowman. Articles for publication should b?- received by
the editor at least three weeks previous to the first of the month on which the
number in which it is designed for them to appear, is issued.
|Cgf"The .American Journal of Dqntal Science will contain fifty pages
of reading matter in each number, making a volume of about
SiHundred pagee,
EXCLUSIVE OF ADVERTISEMENTS.
THE
AMERICAN JOURNAL
OF
DENTAL SCIENCE.
. The Journal is issued on the first of each month and contains not less
than fifty pages of reading matter in each number, exclusive of adver-
tisements, making a volume of six hundred pages per annum.
In each number will be found Original Communications, Corres-
pondence, Selected Articles, Monthly Summary, Bibliographical No-
tices and Editorial Matter, and every effort will be exerted to render
the Journal worthy of the patronage of the Dental profession.
A generous co-operation is respectfully solicited from the members
of the profession ; and as the Journal has now a printing office of it s
own, which materially lessens the cost of its publication, it will be
issued at the reduced subscription price of
$2.SO Per Annum in Advance.
Communications are respectfully solicited on all subjects pertain-
ing to the practice of dentistry, as well as reports of cases occurring
in dental practice.
1 Page.
RATES OF ADVERTISING.
1 MO. 2 MO. 3 MO.
$15 00
i ? | 10 00
i " I 7 00
1 ? ? 5 00
$22 50
15 00
11 00
8 00
$30 00
20 00
15 00
11 00
$50 00
30 00
20 00
15 00
6 MO. i 12 MO.
All original communications designed for publication, works for
review, new instruments for notice, exchanges, &c., should be directed
fo the editor, 259 N Eutaw St., Baltimore, Md.
All advertisements and subscriptions should be addressed t,o
SNOWDEN & COWMAN,
Publishers of the Am. Journal of Dental Science.
86 W. Fayette St. Bet. Charles & Liberty Sts.,
BALTIMORE, MI)

				

